# Evaluation of Cell Responses of *Saccharomyces cerevisiae* under Cultivation Using Wheat Bran as a Nutrient Resource by Analyses of Growth Activities and Comprehensive Gene Transcription Levels

**DOI:** 10.3390/microorganisms11112674

**Published:** 2023-10-31

**Authors:** Akihito Nakanishi, Minori Mori, Naotaka Yamamoto, Shintaro Nemoto, Nono Kanamaru, Misaki Yomogita, Natsumi Omino, Riri Matsumoto

**Affiliations:** 1 School of Bioscience and Biotechnology, Tokyo University of Technology, Tokyo 192-0982, Japan; b0a20160a1@edu.teu.ac.jp (M.M.); b0a2005162@edu.teu.ac.jp (N.K.); b0a20043f8@edu.teu.ac.jp (N.O.);; 2 Graduate School of Bionics, Tokyo University of Technology, Tokyo 192-0982, Japan; g11220402a@edu.teu.ac.jp (N.Y.); g1123026c5@edu.teu.ac.jp (S.N.); g112204255@edu.teu.ac.jp (M.Y.)

**Keywords:** wheat bran, yeast cultivation, cell response, transcriptome

## Abstract

Wheat bran has high nutritional values and is also cheaper than yeast nitrogen base as an important component of a medium. Although its use in microbial cultivations is expected, research and development has hardly progressed so far. In this study, with experimental *Saccharomyces cerevisiae* BY4741, the cell responses to wheat bran as a nutrient were evaluated by analyses of cell growth, ethanol production, and comprehensive gene transcription levels. Comparing wheat bran and yeast nitrogen base, BY4741 showed specific growth rates of 0.277 ± 0.002 and 0.407 ± 0.035 as a significant difference. Additionally, wheat bran could be used as a restricted media component like yeast nitrogen base. However, in 24 h of cultivation with wheat bran and yeast nitrogen base, although conversion ratios of ethanol productions showed no significant difference at 63.0 ± 7.2% and 62.5 ± 8.2%, the ratio of cell production displayed a significant difference at 7.31 ± 0.04% and 4.90 ± 0.16%, indicating a different cell response. In fact, the comprehensive evaluation of transcription levels strongly suggested major changes in glucose metabolism. This study indicated that BY4741 could switch transcription levels efficiently to use wheat bran.

## 1. Introduction

Yeast is a very important eukaryotic microorganism in the food industry because of its excellent ability to ferment carbohydrates [1]. Among them, the budding yeast *Saccharomyces cerevisiae* is highly safe as a microorganism that has been used in fermentation production of bread, sake, and beer since ancient times [1]. Therefore, *S. cerevisiae* is a familiar strain to industry, so that many studies have been enthusiastically conducted on its life cycles [2,3], genetic engineering [4], the controlling ploidy such as haploid and diploid [5], and improving the activities of its growth and fermentation [6]. In particular, BY4741 and BY4742, prepared as experimental strains of *S. cerevisiae,* are haploid, so the effects of the introduced genes are readily expressed in the phenotype [7]; those strains have *his3*, *leu2*, *met15,* and *ura3* deleted as the essential genes for life maintenance [8], so that those are used in genetic engineering as the nutritional requirement markers, with the result that there are many reports of the creation of mutants by using the plasmid vector system and genome integration system and of the evaluation of the activities of those strains [9]. Although many reports regarding those experimental strains do indeed exist, traditional selective media are still mostly used [10]. A commonly used selection medium for *S. cerevisiae* strains like BY4741 is synthetic dextrose medium (SD medium with essential nutrients (YG-group medium, as it is termed in this study)), which contains glucose as a carbon source, yeast nitrogen base without amino acid (YNB) as a nutrient source, and other elements to meet the nutritional requirements of each *S. cerevisiae* genotype [10]. However, this synthetic medium is expensive, and YNB, which is a constituent nutrient, is extremely expensive. As of August 2023, the YNBs were 14,096 JPY per 100 g by Funakoshi Co., Ltd. (Tokyo, Japan), 16,500 JPY by Merck KGaA (Darmstadt, Germany), and 15,600 JPY by Nacalai Tesque Inc. (Kyoto, Japan). YNB popularly contains salts (KH_2_PO_4_ 1000 mg·L^−1^; MgSO_4_ 500 mg·L^−1^; NaCl 100 mg·L^−1^; CaCl_2_ 100 mg·L^−1^), vitamins (biotin 0.002 mg·L^−1^; Ca pantothenate 0.4 mg·L^−1^; folic acid 0.002 mg·L^−1^; inositol 2.0 mg·L^−1^; niacin 0.4 mg·L^−1^; PABA 0.2 mg·L^−1^; pyridoxine-HCl 0.4 mg·L^−1^; riboflavin 0.2 mg·L^−1^; thiamine-HCl 0.4 mg·L^−1^), and trace elements (boric acid 0.5 mg·L^−1^; CuSO_4_ 0.04 mg·L^−1^; KI 0.1 mg·L^−1^; FeCl_3_ 0.2 mg·L^−1^; MnSO_4_ 0.4 mg·L^−1^; Na_2_MoO_4_ 0.2 mg·L^−1^; ZnSO_4_ 0.4 mg·L^−1^) [11], and replacing YNB with cheaper nutrient sources could greatly reduce the economic cost of experimentation with *S. cerevisiae* strains like BY4741. Food residues containing various elements are powerful nutrient sources for cultivating *S. cerevisiae*, and wheat bran, which is rich in nutrient sources such as vitamins and minerals, was focused on in this study. In addition, wheat bran has an advantage in price [12,13]. Moreover, if wheat bran could be used as a nutrient source in a restricted medium instead of YNB, the cost of culturing experimental *S. cerevisiae* strains such as BY4741 in the restricted medium could be greatly reduced; however, there have been few reports regarding the use of wheat bran as a nutrient, meaning that the cell responses of *S. cerevisiae* in medium containing wheat bran have not been clear. Therefore, the investigation of the effects on *S. cerevisiae* of the use of wheat bran as a nutrient source has significance for the field in microorganisms. In the use of wheat bran, the evaluation of the effects on fermentation by *S. cerevisiae* especially should be deeply analyzed because *S. cerevisiae* has fermentable strains and their fermentability would be important in the food industry. Regarding the fermentation by *S. cerevisiae* using food residues, for instance, the ethanol production by *S. cerevisiae* was 49.5 g·L^−1^ using 100 g·L^−1^ of instant noodle waste as a nutrient source [14] and 21 g·L^−1^ using 69 g·L^−1^ of reducing sugar derived from potato peel [15], indicating the differences in fermentability due to differences in substrates. Additionally, the cells are possibly caused to switch the metabolic flow depending on the gene transcription levels in the medium containing wheat bran, so that the comprehensively shifted transcription levels as direct cell responses should be as the subject of transcriptome analysis. Transcriptome analysis is a powerful assessment tool to understand how the strains tend to alter their metabolic activities [16], and can reveal how strains tend to switch their metabolic response due to using the food residues. Recently, as a value-added substance-producing strain, mutants of *S. cerevisiae* have been created to produce those substances instead of ethanol [17], and in particular, genetically modified strains were used to produce various substances such as butanol [18] and amino acids [19]. Therefore, fermentation should be evaluated in the use of the food residues from the viewpoint of not only production of several substances but also the transcription levels relating to that production in broad areas. To date, using food residues, although the evaluation of the fermentative production by *S. cerevisiae* has been reported many researchers [20,21,22], there are few reports of detailed evaluation of the cell responses by transcriptome analysis. The evaluation of the cell responses from the viewpoint of transcriptome analysis could have a significant impact, in the sense that it could lead to controlling intracellular metabolism.

In this study, the laboratory yeast *S. cerevisiae* BY4741 was selected as a targeted strain, and cultured in a wheat bran-fed medium to determine whether wheat bran could be used as a medium component (Table 1). At that time, the cell responses were evaluated by analyses of cell proliferation, growth under nutrient limitation, the production of cells and ethanol, and transcriptomic analysis. This study finally showed the possibility of wheat bran as an alternative nutrient to YNB especially as a templated medium component for *S. cerevisiae*, indicating the reduction in research costs and the expansion of substance-producing possibilities. Therefore, the study of using wheat bran as the nutrient for yeast fermentation could demonstrate an industrial impact.

**Table 1:** List of medium compositions.

Medium Name	Main Component		Essential Nutrients				Corresponding Figure
WG+HLMU medium	wheat bran	glucose	histidine	leucine	methionine	uracil	Figure 1, Figure 2, Figure 3, Figure 4, Figure 5 and Figure 6
W+HLMU medium	wheat bran		histidine	leucine	methionine	uracil	Figure 1
WG+LMU medium	wheat bran	glucose		leucine	methionine	uracil	Figure 2
WG+HMU medium	wheat bran	glucose	histidine		methionine	uracil	Figure 2
WG+HLU medium	wheat bran	glucose	histidine	leucine		uracil	Figure 2
WG+HLM medium	wheat bran	glucose	histidine	leucine	methionine		Figure 2
YG+HLMU medium (SD medium)	YNB	glucose	histidine	leucine	methionine	uracil	Figure 1, Figure 3, Figure 4, Figure 5 and Figure 6
Y+HLMU medium	YNB		histidine	leucine	methionine	uracil	Figure 1
G+HLMU medium		glucose	histidine	leucine	methionine	uracil	Figure 1
HLMU medium			histidine	leucine	methionine	uracil	Figure 1

**Figure 1:**
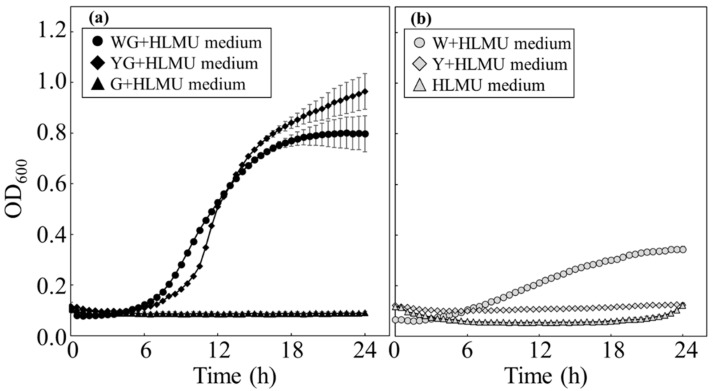
Time course profiles of growth of BY4741 in each medium. BY4741 was cultivated to evaluate the growth manners in each medium. (**a**) With glucose, BY4741 was cultured in WG+HLMU medium (●), in YG+HLMU medium (◆), and in G+HLMU medium (▲). (**b**) Without glucose, BY4741 was cultured in W+HLMU medium (○), in Y+HLMU medium (◇), and in HLMU medium (△). (*n* = 6~12).

**Figure 2:**
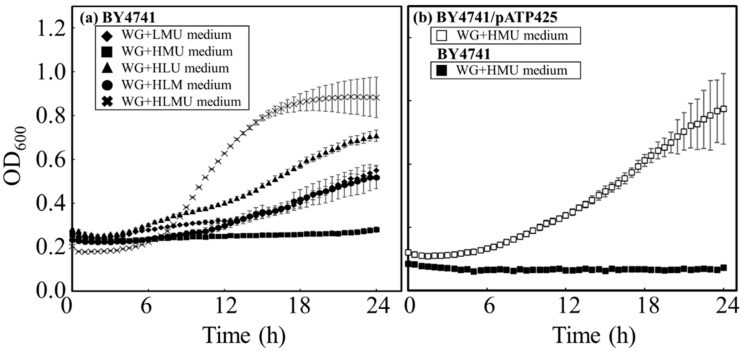
Time course profiles of growth of BY4741 and BY4741/pATP425 in each restricted medium. BY4741 was cultured to evaluate cell usability in each restricted medium prepared with wheat bran. (**a**) BY4741 was cultured in wheat bran media without histidine (◆: WG+LMU medium), without leucine (◼: WG+HMU medium), without methionine (▲: WG+HLU medium), without uracil (●: WG+HLM medium), and with nothing missing (×: WG+HLMU medium) (n = 6~12). (**b**) BY4741/pATP425 was cultured in wheat bran media without leucine (□: WG+HMU medium) and BY4741 was also cultured in wheat bran media without leucine (◼: WG+HMU medium) (*n* = 6~12).

**Figure 3:**
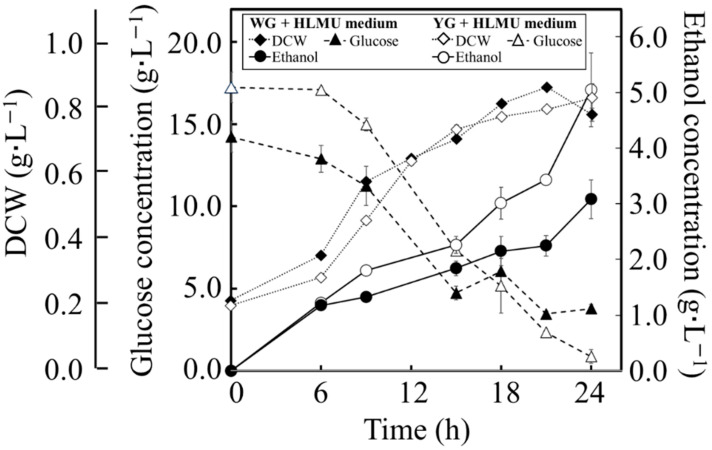
Time course profiles of fermentation by BY4741 in each medium. BY4741 was cultured in WG+HLMU medium and YG+HLMU medium to evaluate DCW as cell production (◆: in WG+HLMU medium; ◇: in YG+HLMU medium), glucose concentration as glucose consumption (▲: in WG+HLMU medium; △: in YG+HLMU medium), and ethanol concentration as ethanol production (●: in WG+HLMU medium; ○: in YG+HLMU medium) over time (*n* = 6~12).

**Figure 4:**
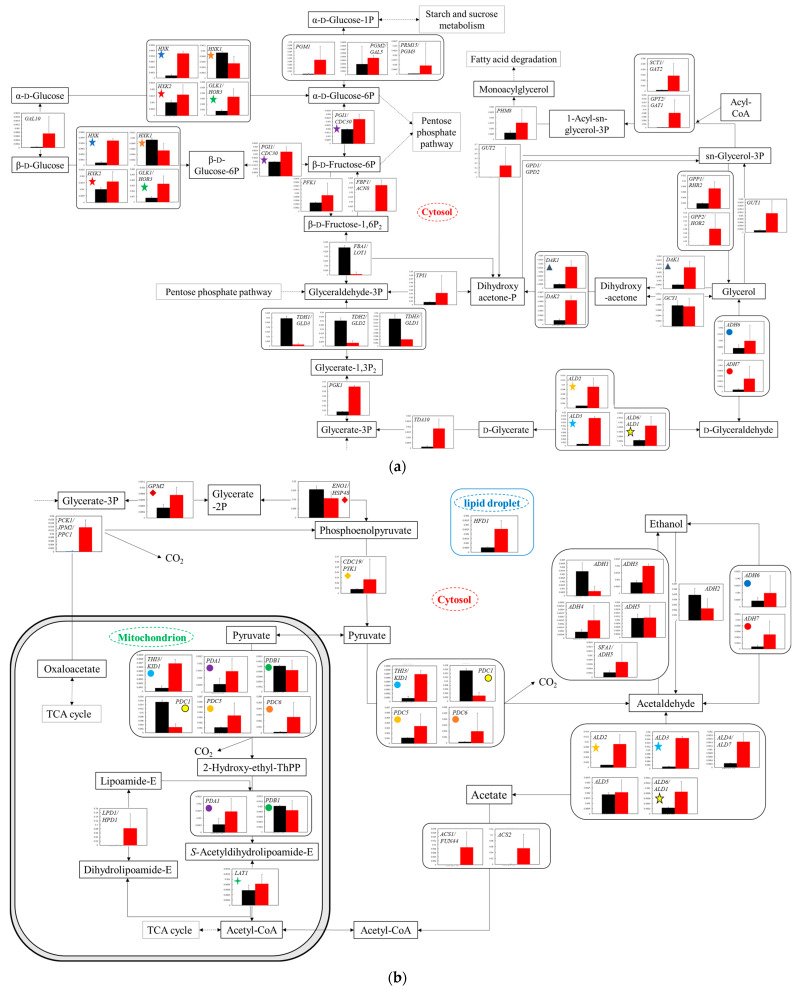
Comparison of gene transcription levels in YG+HLMU medium and WG+HLMU medium in glycolysis. Data are shown as relative mRNA transcription levels normalized by the level of *RDN18* as a housekeeping gene. Relative transcription levels in YG+HLMU medium and WG+HLMU medium at 15–20 h of main fermentation are shown in black and red, respectively. Error bars indicate SD of 3~8-time replicates experiments. Gene abbreviations in (**a**): *ADH6*: *NADP-dependent alcohol dehydrogenase 6*, *ADH7*: *NADP-dependent alcohol dehydrogenase 7*, *ALD2*: *aldehyde dehydrogenase (NAD^+^) 2*, *ALD3*: *aldehyde dehydrogenase (NAD^+^) 3*, *ALD6/ADL1*: *aldehyde dehydrogenase (NADP^+^)/aldehyde dehydrogenase (NAD^+^) 6*, *DAK1*: *dihydroxyacetone kinase/FAD-AMP lyase 1*, *DAK2*: *dihydroxyacetone kinase/FAD-AMP lyase 2*, *FBA1/LOT1*: *fructose-bisphosphate aldolase*, *FBP1/ACN8*: *fructose 1*,*6-bisphosphate 1-phosphatase*, *GAL10*: *UDP-glucose 4-epimerase aldose 1-epimerase*, *GCY1*: *glycerol 2-dehydrogenase (NADP^+^)*, *GLK1/HOR3*: *hexokinase*, *GPP1/RHR2*: *glycerol-1-phosphatase 1*, *GPP2/HOR2*: *glycerol-1-phosphatase 2*, *GPT2/GAT1*: *glycerol-3-phosphate O-acyltransferase/dihydroxyacetone phosphate acyltransferase*, *GUT1*: *glycerol kinase1*, *GUT2*: *glycerol kinase 2*, *HXK*: *hexokinase*, *HXK1*: *hexokinase 1*, *HXK2*: *hexokinase 2*, *PFK1*: *fructose-bisphosphate aldolase*, *PGK1*: *phosphoglycerate kinase*, *PGI1/CDC30*: *glucose-6-phosphate isomerase*, *PGM1*: *UDP-glucose 4-epimerase aldose 1-epimerase*, *PGM2/GAL5*: *phosphoglucomutase 2*, *PHM8*: *2-lysophosphatidate phosphatase*, *PRM15/PGM3*: *phosphoribomutase 3*. *SCT1/GAT2*: *glycerol-3-phosphate O-acyltransferase/dihydroxyacetone phosphate acyltransferase 2*, *TDA10*: *putative ATP-dependent kinase/d-glycerate 3-kinase*, *TDH1/GLD3*: *glyceraldehyde-3-phosphate dehydrogenase 1*, *TDH2/GLD2*: *glyceraldehyde-3-phosphate dehydrogenase 2*, *TDH3/GLD1*: *glyceraldehyde-3-phosphate dehydrogenase 3*, *TPI1*: *triose-phosphate isomerase 1*. In (**b**): *ACS1/FUN44*: *acetyl-CoA synthetase 1*, *ACS2*: *acetyl-CoA synthetase 2*, *ADH1*: *alcohol dehydrogenase 1*, *ADH2*: *alcohol dehydrogenase 2*, *ADH3*: *alcohol dehydrogenase 3*, *ADH4*: *alcohol dehydrogenase 4*, *ADH5*: *alcohol dehydrogenase 5*, *ALD4/ADL7*: *aldehyde dehydrogenase (NADP^+^)*, *ALD5*: *aldehyde dehydrogenase (NAD(P)^+^) 5*, *CDC19/PYK1*: *pyruvate kinase*, *ENO1/HSP48*: *phosphopyruvate hydratase*, *GPM2*: *phosphoglycerate mutase*, *HFD1*: *hexadecenal dehydrogenase/aldehyde dehydrogenase (NAD^+^)*, *LAT1*: *pyruvate dehydrogenase E2 component*, *LPD1/HPD1*: *dihydrolipoyl dehydrogenase*, *PCK1/JPM2/PPC1*: *phosphoenolpyruvate carboxykinase*, *PDA1*: *pyruvate dehydrogenase E1 component subunit alpha*, *PDB1*: *pyruvate dehydrogenase E1 component subunit beta*, *PDC1*: *pyruvate decarboxylase 1*, *PDC5*: *pyruvate decarboxylase 5*, *PDC6*: *pyruvate decarboxylase 6*, *SFA1/ADH5*: s*-(hydroxymethyl)glutathione dehydrogenase/alcohol dehydrogenase*, *THI3/KID1*: *pyruvate decarboxylase*. Similarly colored and shaped marks in figures mean transcription levels of the same enzyme genes. (**a**) Comparison of gene transcription levels in YG+HLMU medium and WG+HLMU medium on upstream of glycolysis. (**b**) Comparison of gene transcription levels in YG+HLMU medium and WG+HLMU medium on downstream of glycolysis and partial reactions in mitochondrion.

**Figure 5:**
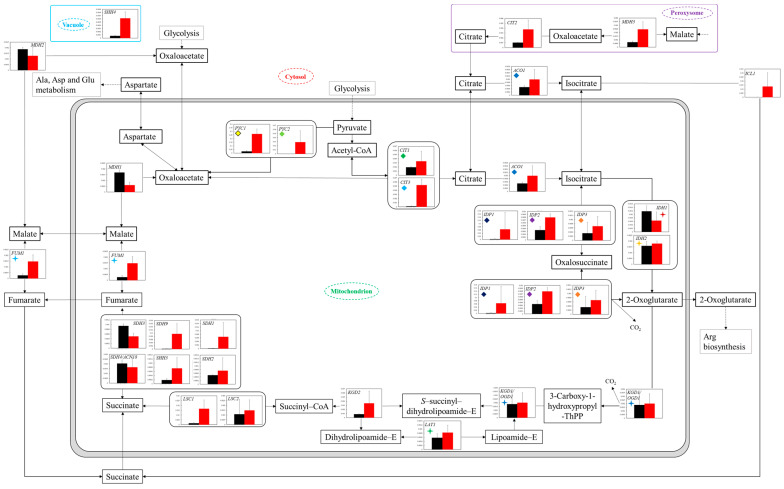
Comparison of gene transcription levels in YG+HLMU medium and WG+HLMU medium on TCA cycle and relating reactions. Data are shown as relative mRNA transcription levels normalized by the level of *RDN18* as a housekeeping gene. Relative transcription levels in YG+HLMU medium and WG+HLMU medium at 15–20 h of main fermentation are shown in black and red, respectively. Error bars indicate SD of 3~8-time replicates experiments. Gene abbreviations in Figure 5: *ACO1*: *aconitate hydratase*, *CIT1*: *citrate synthase 1*, *CIT2*: *citrate synthase 2*, *CIT3*: *citrate synthase 3*, *FUM1*: *fumarate hydratase*, *class II*, *IDH1*: *isocitrate dehydrogenase (NAD^+^) 1*, *IDH2*: *isocitrate dehydrogenase (NAD^+^) 2*, *IDP1*: *isocitrate dehydrogenase 1*, *IDP2*: *isocitrate dehydrogenase 2*, *IDP3*: *isocitrate dehydrogenase 3*, *KGD1/OGD1*: *2-oxoglutarate dehydrogenase E1 component*, *KGD2*: *2-oxoglutarate dehydrogenase E2 component (dihydrolipoamide succinyltransferase)*, *LSC1*: *succinyl-CoA synthetase alpha subunit*, *LSC2*: *succinyl-CoA synthetase beta subunit*, *MDH1*: *malate dehydrogenase 1*, *MDH2*: *malate dehydrogenase 2*, *MDH3*: *malate dehydrogenase 3*, *PYC1*: *pyruvate carboxylase 1*, *PYC2*: *pyruvate carboxylase 2*, *SDH1*: *succinate dehydrogenase (ubiquinone) flavoprotein subunit*, *SDH2*: *succinate dehydrogenase (ubiquinone) iron-sulfur subunit*, *SDH3*: *succinate dehydrogenase (ubiquinone) cytochrome b560 subunit*, *SDH4/ACN18*: *succinate dehydrogenase (ubiquinone) membrane anchor subunit*, *SDH9*: *succinate dehydrogenase (ubiquinone) flavoprotein subunit*, *SHH3*: *succinate dehydrogenase (ubiquinone) cytochrome b560 subunit*, *SHH4*: *succinate dehydrogenase (ubiquinone) membrane anchor subunit*. Similarly colored and shaped marks in figures mean transcription levels of the same enzyme genes.

**Figure 6:**
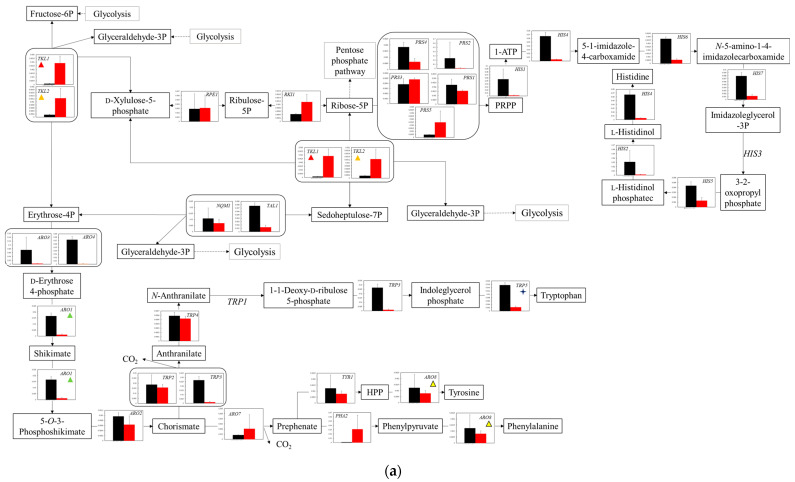

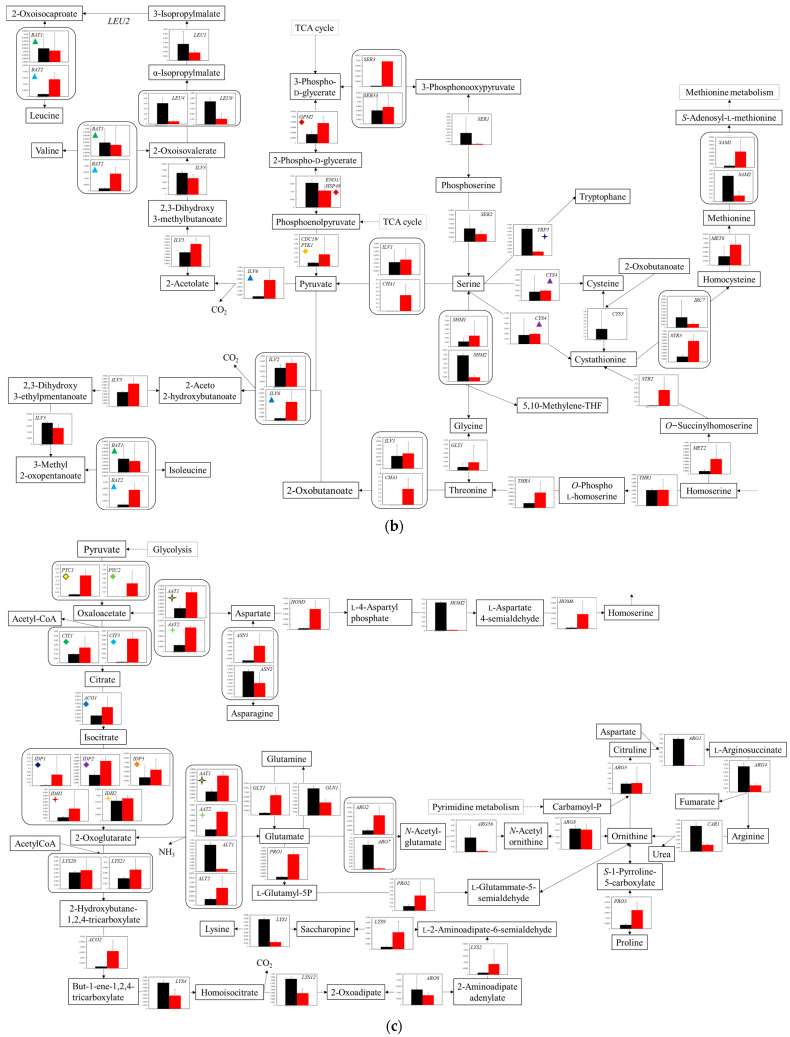
Comparison of gene transcription levels in YG+HLMU medium and WG+HLMU medium on biosynthesis of amino acids. Data are shown as relative mRNA transcription levels normalized by the level of *RDN18* as a housekeeping gene. Relative transcription levels in YG+HLMU medium and WG+HLMU medium at 15–20 h of main fermentation are shown in black and red, respectively. Error bars indicate SD of 3~8-time replicates experiments. Gene abbreviations in (**a**): *ARO1*: *3-phosphoshikimate 1-carboxyvinyltransferase*, *ARO2*: *chorismite synthase*, *ARO3*: *3-deoxy-7-phosphoheptulonate synthase 3*, *ARO4*: *3-deoxy-7-phosphoheptulonate synthase 4*, *ARO7*: *chorismite mutase*, *ARO8*: *aromatic amino acid aminotransferase*, *HIS1*: *ATP phosphoribosyltransferase*, *HIS2*: *histidinol-phosphatase*, *HIS4*: *phosphoribosyl-ATP pyrophosphohydrolase*, *HIS5*: *histidinol-phosphate aminotransferase*, *HIS6*: *phosphoribosylformimino-5-aminoimidazole carboxamide ribotide isomerase*, *HIS7*: *imidazole glycerol-phosphate synthase*, *NQM1*: *transaldolase*, *PHA2*: *prephenate dehydratase*, *PRS1*: *ribose 5-phosphate isomerase*, *PRS2*: *ribose-phosphate pyrophosphokinase 2*, *PRS3*: *ribose-phosphate pyrophosphokinase 3*, *PRS4*: *ribose-phosphate pyrophosphokinase 4*, *PRS5*: *ribose-phosphate pyrophosphokinase 5*, *RKI1*: *ribose 5-phosphate isomerase*, *RPE1*: *ribulose-phosphate 3-epimerase*, *TAL1*: *transaldolase*, *TKL1*: *transketolase 1*, *TKL2*: *transketolase 2*, *TRP2*: *anthranilate synthase*, *TRP3*: *anthranilate synthase*/*indole-3-glycerol-phosphate synthase*, *TRP4*: *anthranilate phosphoribosyltransferase*, *TRP5*: *tryptophan synthase*, *TYR1*: *prephenate dehydrogenase*. In (**b**): *BAT1*: *branched-chain amino acid aminotransferase 1*, *BAT2*: *branched-chain amino acid aminotransferase 2*, *CHA1*: *l-serine/l-threonine ammonia-lyase*, *CYS3*: *cystathionine gamma-lyase*, *CYS4*: *cysteine synthase*, *GLY1*: *threonine aldolase*, *ILV1*: *l-serine/l-threonine ammonia-lyase*, *ILV2*: *acetolactate synthase catalytic subunit 2*, *ILV3*: *dihydroxy-acid dehydratase*, *ILV5*: *ketol-acid reductoisomerase*, *ILV6*: *acetolactate synthase*, *IRC7*: *cysteine-S-conjugate beta-lyase*, *LEU1*: *3-isopropylmalate dehydratase*, *LEU4*: *2-isopropylmalate synthase 4*, *LEU9*: *2-isopropylmalate synthase 9*, *MET2*: *homoserine O-acetyltransferase*, *MET6*: *5-methyltetrahydropteroytriglutamate-homocysteine methyltransferase*, *SAM1*: *s-adenosylmethionine synthetase1*, *SAM2*: *s-adenosylmethionine synthetase2*, *SER1*: *phosphoserine aminotransferase*, *SER2*: *phosphoserine phosphatase*, *SER3*: *d-3-phosphoglycerate dehydrogenase 3*, *SER33*: *d-3-phosphoglycerate dehydrogenase 33*, *SHM1*: *glycine hydroxymethltransferase1*, *SHM2*: *glycine hydroxymethltransferase2*, *STR2*: *cystathionine gamma-synthase*, *STR3*: *cysteine-S-conjugate beta-lyase*, *THR1*: *homoserine kinase*, *THR4*: *threonine synthase*. In (**c**): *AAT1*: *alanine transaminase*, *AAT1*: *aspartate aminotransferase*, *AAT2*: *alanine transaminase*, *AAT2*: *aspartate aminotransferase*, *ACO2*: *homoaconitase*, *ALT1*: *alanine transaminase 1*, *ALT2*: *alanine transaminase 2*, *ARG1*: *argininosuccinate synthase*, *ARG2*: *amino-acid N-acetyltransferase*, *ARG3*: *ornithine carbamoyltransferase*, *ARG4*: *argininosuccinate lyase*, *ARG7*: *amino-acid N-acetyltransferase*, *ARG8*: *glutamate N-acetyltransferase*, *ARG56*: *N-acetyl-gamma-glutamyl-phosphate reductase*, *ASN1*: *asparagine synthase 1*, *ASN2*: *asparagine synthase 2*, *CAR1*: *arginase*, *GLT1*: *glutamate synthase*, *GLN1*: *glutamine synthetase*, *HOM2*: *aspartate-semialdehyde dehydrogenase*, *HOM3*: *aspartate kinase*, *HOM6*: *homoserine dehydrogenase*, *LYS1*: *saccharopine dehydrogenase 1*, *LYS2*: *l-2-aminoadipate reductase*, *LYS4*: *homoaconitate hydratase*, *LYS9*: *saccharopine dehydrogenase 9*, *LYS12*: *homoisocitrate dehydrogenase*, *LYS20*: *homocitrate synthase 20*, *LYS21*: *homocitrate synthase 21*, *PRO1*: *glutamate 5-kinase*, *PRO2*: *glutamate-5-semialdehyde dehydrogenase*, *PRO3*: *pyrroline-5-carboxylate reductase*. The similarly colored and shaped marks in figures mean transcription levels of the same enzyme genes. (**a**) Comparison of gene transcription levels in YG+HLMU medium and WG+HLMU medium for biosynthesis of amino acids relating to pentose phosphate pathway. (**b**) Comparison of gene transcription levels in YG+HLMU medium and WG+HLMU medium for biosynthesis of amino acids from pyruvate. (**c**) Comparison of gene transcription levels in YG+HLMU medium and WG+HLMU medium for biosynthesis of amino acids relating to pyruvate and TCA cycle.

## 2. Materials and Methods

### 2.1. Strains and Medium-Conditions

*Saccharomyces cerevisiae* BY4741 strain (*MATa*, *his3Δ1*, *leu2Δ0*, *met15Δ0*, *ura3Δ0*) [8] was purchased from Funakoshi as a laboratory haploid strain. BY4741 was transformed with pATP425 as a leucine-supplying plasmid [23,24] by the lithium acetate method [25], and then BY4741/pATP425. YG-group media were prepared as 20 g·L^−1^ glucose, 6.7 g·L^−1^ YNB, and essential nutrients (20 mg·L^−1^ histidine, 120 mg·L^−1^ leucine, 20 mg·L^−1^ methionine, and 20 mg·L^−1^ uracil). WG-group media were prepared with wheat bran purchased from Oriental Yeast Co., Ltd. (Tokyo, Japan) as a nutrient source instead of YNB, and the wheat bran concentration was 6.7 g·L^−1^. The purpose of culturing with wheat bran in this study was to evaluate the cell response when wheat bran was substituted for YNB. Therefore, the final concentration of wheat bran in the medium was adjusted only to 6.7 g∙L^−1^, which was the same as the final concentration of YNB. The restricted media based on media of WG-group lacked one of the essential nutrients as shown, 20 mg·L^−1^ histidine, 120 mg·L^−1^ leucine, 20 mg·L^−1^ methionine, or 20 mg·L^−1^ uracil. Information on each medium is shown in Table 1. The component information of wheat bran was obtained from elemental analysis by Japan food research laboratories (Tokyo, Japan) and that of YNB was from the company database [11].

### 2.2. Evaluation of Relative Growth Rate and Doubling Time in Each Medium

Cell responses of *S. cerevisiae* in each medium condition were analyzed as shown below. Optical density at 600 nm (OD_600_) was measured to quantify the strain cells. To analyze the growth activities of *S. cerevisiae*, 200 µL of *S. cerevisiae* broth was cultivated in each well of a 96-well plate under a strong vibration mode (5 s, strong mixing mode) in each 30 min at 23 °C as room temperature, and monitored at OD_600_ with a 96-well microplate reader SH-1300Lab (Hitachi High-Tech Science Co., Tokyo, Japan). The initial OD_600_ was calibrated to approximately 0.1~0.3. Relative growth rate (*µ*) and doubling time (*td*) were calculated by the formulas below (*t*_1_: sampling time 1 (*h*); *t*_2_: sampling time 2 (*h*) (*t*_2_ ˃ *t*_1_); *S*_1_: scale of concentration indicated by OD_600_ at *t*_1_; *S*_2_: scale of concentration indicated by OD_600_ at *t*_2_).

$$µ=\frac{ln(S_{2}/S_{1})}{\left(t_{2}-t_{1}\right)}\;(h^{-1})$$

$$td=\frac{ln2}{µmax}\;(h)$$

### 2.3. Evaluation of Concentrations of Glucose, Ethanol, and General Media Components (Carbohydrate, Lipid, and Protein)

Glucose and ethanol concentration were quantified using a high-performance liquid chromatography (HPLC) system after cultivation and pre-preparation. For cultivation, 10 mL of *S. cerevisiae* broth was cultivated with 130 strokes per minute (spm) at 30 °C, and also monitored at OD_600_ with a U-2900 (Hitachi High-Technologies Co., Tokyo, Japan). The initial OD_600_ was calibrated to approximately 0.1. As pre-preparation, 1 mL of each broth was collected at the middle of the exponential phase, and the supernatant was obtained by centrifugation at 5000× *g* for 1 min. The supernatant was filtered with a Millex^®^ syringe filter (pore size: 0.45 µm) (Merck KGaA), and the flow-through was analyzed with the HPLC system. The HPLC system consisted of a LC-20AD pump (Shimadzu Co., Kyoto, Japan), a CTO-10A column oven (Shimadzu Co.), an SPD-20AV detector (Shimadzu Co.), a RezexTM RHM-Monosaccharide H^+^ column (300 × 7.8 mm) (Phenomenex Inc., Tokyo, Japan), and a 7725 injector (Rheodyne Inc., Bensheim, Germany). The concentrations of glucose and ethanol were identified and quantified at 190 nm, with chromatographic data monitored by the SPD-20AV detector. The mobile phase was water, the column oven was set at 80 °C, and the flow rate was 0.6 mL·min^−1^.

Carbohydrate concentration, lipid concentration, and protein concentration were evaluated by the methods shown below. Evaluation of total carbohydrate quantity: the carbohydrate in the medium was quantified by the anthrone method [26,27]. An object was added to 0.004% (*w*/*v*) anthrone in 75% H_2_SO_4_ on ice and stirred well. The mixture was then placed in a block incubator at 100 °C for 15 min. After 10 min on ice, the mixture was stirred well via vortexing. The correlation between absorbance at 620 nm and carbohydrate content was indicated by a calibration curve that was prepared with glucose, and the supernatant in the reaction was quantitatively evaluated with the calibration curve after a 20-fold dilution. Evaluation of total lipid quantity: lipids in the medium supernatant were methyl esterified with a fatty acid methylation kit (Nacalai Tesque Inc.) [28,29]. The fatty acid methyl esters were identified and quantified with a capillary gas chromatograph GC-2025 (Shimadzu Co., Kyoto, Japan), which was equipped with a DB-23 capillary column (60 m, 0.25 mm internal diameter, 0.15 μm film thickness) (Agilent Technologies Ltd., Santa Clara, CA, USA) and the previous method was then followed [29]. Heptadecanoic acid (Sigma-Aldrich Co., St. Louis, MO, USA) was used as an internal standard, and rapeseed oil (Merck KgaA) was used as a quantitative standard. Evaluation of total protein quantity: the protein in the medium was quantified using a bicinchoninic acid protein assay kit (Takara Bio Inc., Shiga, Japan).

### 2.4. Evaluation of Consumption (Glucose) and Products (Cells and Ethanol) as Fermentation Results

To evaluate glucose consumption in BY4741 fermentation, the concentration of glucose in the broth (g·L^−1^) was obtained by using the HPLC system explained in Section 2.3. The concentration of totally consumed glucose (g·L^−1^) was obtained by subtracting the glucose concentration (g·L^−1^) at the time of interest from the glucose concentration (g·L^−1^) at 0 h.

To evaluate cell production as a product, firstly, the cell density (g·L^−1^) was obtained from the value of OD_600_ by using the calibration curve between OD_600_ and dry-cell weight (DCW) (g·L^−1^): DCW (g·L^−1^) = 0.245 × OD_600_ + 0.153. Secondly, the value of 0.47 × DCW is equal to the weight of the carbon component of the cells [5], and the weight of the carbon component of the cells was obtained from 0.47 × DCW. Finally, the concentration of the glucose consumed for cell production was found by dividing the weight of the carbon by 0.4 (i.e., (6 × 12 (g∙mol^−1^))/180 (g∙mol^−1^)). The conversion ratio of cell production from glucose was calculated as a percentage of the value by dividing the amount of glucose used for cell production by the total amount of glucose consumption.

To evaluate ethanol production as a product, the concentration of the ethanol produced as fermentation (g·L^−1^) was also found by using the HPLC system explained in Section 2.3. The concentration of the consumed glucose in the medium is equal to 1.96 times the weight of the produced ethanol (g·L^−1^) because 1.96 times the weight of ethanol produced in fermentation (i.e., 180 (g∙mol^−1^)/(2 × 46 (g∙mol^−1^))) corresponds to the weight of the consumed glucose [30]. The conversion ratio of ethanol production from glucose was calculated as a percentage of the value by dividing the amount of glucose used for ethanol production by the total amount of glucose consumption. The conversion ratio of ethanol was equal to the theoretical yield given by Feist et al. [31].

### 2.5. Measurement of Gene Translation Levels

*S. cerevisiae* was cultivated in each medium (WG+HLMU medium; YG+HLMU medium) with 130 spm at 30 °C. Approximately 5.0 mg of cells was collected, estimated by OD_600_-the dry-cell weight calibration curve, by centrifugation at 21,500 × *g* for 5 min. The collected strains were mixed with 50 µL of QIAzol Lysis Reagent (QIAGEN GmBH, Tokyo, Japan) and shaken at 23 °C for 5 min. After shaking, 10 µL of chloroform was added and placed on ice for 3 min. The treated samples were centrifuged at 21,500× *g* for 15 min; the supernatant was shaken with 25 µL of isopropanol; the mixture was placed at 23 °C for 10 min. The supernatant was discarded after centrifugation at 21,500× *g* for 10 min and then the precipitant was rinsed with 1 mL of 70% ethanol. The rinsed sample was dried with lyophilizer Refrigerated CentriVap Benchtop Vacuum Concentrator (Labconco Corp., Kansas City, MO, USA) and the dried precipitant was dissolved in 10 µL of Rnase-free water. The prepared sample as total RNA was used to synthesize complementary DNA (cDNA) using a ReverTra Ace qPCR RT Master Mix with gDNA Remover (TOYOBO Co., Ltd., Osaka, Japan). With the cDNA, quantitative PCR was performed using Mx qPCR Systems (Agilent Technologies Ltd., Santa Clara, CA, USA) with THUNDERBIRD SYBR qPCR Mix (TOYOBO Co., Ltd.). The PCR cycle was as follows: (1) initial denaturation at 95 °C for 2 min; (2) 40 cycles of further denaturation at 95 °C for 3 s, annealing at 60 °C for 30 s; (3) melting curve analysis at 95 °C for 15 s, at 60 °C for 1 min, and at 95 °C for 15 s. The average threshold cycle values were evaluated throughout the logarithmic amplification phase, and were normalized by the level of *RDN18*. The quantitative PCR primers (Supplemental Table S1) were designed using the Primer3Plus algorithm (https://dev.primer3plus.com/index.html (accessed on 12/10/2022)), based on information of each predicted gene sequence from the genome information of NCBI.

## 3. Results

### 3.1. Use of Wheat Bran as a Medium Component for BY4741 Cell Growth

To evaluate whether the wheat bran could be used as a substitute for YNB, *S. cerevisiae* was cultured in WG+HLMU medium (Figure 1a). As the result, the growth profiles in the media of WG+HLMU medium and YG+HLMU medium showed 0.80 ± 0.07 and 0.96 ± 0.04 of the OD_600_ values as the cell density at 24 h, respectively, indicating that the cell density in WG+HLMU medium was lower than that in YG+HLMU medium. Additionally, the *µ*_max_ values in the media of WG+HLMU medium and YG+HLMU medium were 0.277 ± 0.002 in 6.0~9.0 h and 0.407 ± 0.035 in 10.0~11.5 h as the logarithmic growth phases (Table 2), also indicating that the growth rate in WG+HLMU medium was lower than that in YG+HLMU medium, with a significant difference (*p* < 0.01). On the other hand, the values of OD_600_ and *µ*_max_ as the growth profiles in the medium without the wheat bran and YNB did not show an increase in cell growth. In addition, to evaluate whether the wheat bran could be used as a carbon source instead of glucose, *S. cerevisiae* was also cultivated in media excluding glucose (Figure 1b). A few increases of cell density in W+HLMU medium were found, compared to no increase in Y+HLMU medium at 24 h. In addition, no significant difference was experimentally confirmed in the correlation diagrams between the OD_600_ value and cell production (g·L^−1^) and between OD_600_ value and viable cell density (cfu∙mL^−1^) obtained under all medium conditions, with the result that there was no difference in the weight per cell regardless of culture media. *S. cerevisiae* could replace many nutrients of YNB for cell growth with the wheat bran, so the composition of the wheat bran was evaluated by comparing it to that of YNB (Table 3). The results showed that the wheat bran and YNB contained no lipids; the wheat bran contained 6.1 g∙L^−1^ of total protein; the wheat bran also contained 15.1 g∙L^−1^ of total carbohydrate (glucose: 1.7 g∙L^−1^) as the carbon source, unlike YNB. In addition, according to the results of the elemental analysis, essential components for cell growth such as Na, Fe, Ca, Cu, and Zn [32,33,34,35,36] appeared at levels below the detection limit for each mineral, as implied by ‘not detected (N.D.)’ given by Japan food research laboratories in the wheat bran, and the others in the wheat bran were lower than those in YNB, except for Mn.

### 3.2. Use of Wheat Bran as a Component of Restriction Media for BY4741

To evaluate whether WG-group medium can be used as a restriction, *S. cerevisiae* strain BY4741 was grown in WG+LMU medium, WG+HLU medium, WG+HMU medium, and WG+HLM medium, as these media lacked essential nutrients, (Figure 2a). As a result, the *µ*_max_ value was 0.277 ± 0.002 (6.0~9.0 h) in WG+HLMU medium, 0.0536 ± 0.0019 (14.0~22.0 h), 0.0521 ± 0.0007 (4.0~22.0 h), and 0.0484 ± 0.0022 (10.0~22.0 h) in WG+LMU medium, WG+HLU medium, and WG+HLM medium; and N.D. as no growth occurred in WG+HMU medium. Then, to confirm the function of leucine as a selective medium in WG-group medium, BY4741/pATP425 transformed with a plasmid containing the leucine marker [23,24] was cultured in WG+HMU medium (Figure 2b), and the resulting *µ*_max_ value was 0.0901 ± 0.0048 (6.0~16.0 h).

### 3.3. Productions of Cell and Ethanol by BY4741 in Media Containing Wheat Bran

The concentrations of glucose and ethanol in the broth were chronologically quantified to analyze the assimilation and the fermentability of BY4741 in WG+HLMU medium (Figure 3). According to the results, although glucose was consumed in WG+HLMU medium over time, the glucose consumption in WG+HLMU medium was slower than that in the YG+HLMU medium. On the other hand, although ethanol was produced in WG+HLMU medium over time, the ethanol production was also lower than that in the YG+HLMU medium. Then, to evaluate the assimilation property of BY4741 in detail, the conversion activity of BY4741 at 24 h was analyzed through the amounts of glucose consumption, cell production and ethanol production. In the WG+HLMU medium, the amounts of glucose consumption, cell production, and ethanol production were 9.57 ± 0.69 g∙L^−1^, 0.600 ± 0.003 g∙L^−1^, and 3.08 ± 0.35 g∙L^−1^. On the other hand, in the YG+HLMU medium, the amounts of glucose consumption, cell production, and ethanol production were 15.8 ± 0.16 g∙L^−1^, 0.659 ± 0.021 g∙L^−1^, and 5.05 ± 0.66 g∙L^−1^.

### 3.4. Evaluation of Cell Responses of BY4741 in Use of Wheat Bran as a Medium Component with Transcriptomic Analysis

#### 3.4.1. Transcriptomic Analysis of Glycolysis

Although the amounts of intracellular metabolites and proteins were less likely to be expressed as immediate cell responses, the gene transcription levels could be more easily expressed than those [37]. Thus, real-time PCR was performed to assess the cell responses in the different broths of WG+HLMU medium and YG+HLMU medium. According to the analytical results of the transcription levels in upstream glycolysis (Figure 4a), the transcription levels in WG+HLMU medium were overall higher than those in YG+HLMU medium; however, the levels showed characteristic differences between the preparatory and pay-off phases in glycolysis. Specifically, the transcription level of *FBA1*/*LOT1* in WG+HLMU medium was 0.0317 times lower than that in YG+HLMU medium, and the levels of *TDH1*/*GLD3*, *TDH2*/*GLD2* and *TDH3*/*GLD1* in WG+HLMU medium were also 0.0560, 0.125, and 0.250 times lower than those in YG+HLMU medium, respectively.

The gene transcription levels in the range from the downstream of glycolysis to the TCA cycle and to ethanol production were analyzed (Figure 4b). Even using the wheat bran instead of YNB, several transcription levels increased although the levels of the counterpart isozyme genes decreased in each metabolic reaction, with the result that the transcription levels basically showed few significant changes. However, there were some instances where the transcription levels that uniquely controlled a specific metabolic reaction were significantly changed: the levels of *PCK1*/*JPM2*/*PPC1*, a pair of *ACS1/FUN44* and *ACS2* as isozyme genes, a group of *ALD2*, *ALD3*, *ALD4*/*ALD7*, and *ALD6*/*ALD1* as the isozyme genes in WG+HLMU medium were 56.1 (*p* < 0.05), 303 (tendency existed), 106 (tendency existed), 9.34 (*p* < 0.05), 15.7 (*p* < 0.01), 6.11 (*p* < 0.05), and 3.61 (tendency existed) times higher than those in YG+HLMU medium. Conversely, *ADH1*, which is a main ethanol producing enzyme in BY4741, in WG+HLMU medium was 0.189 times lower than that in YG+HLMU medium.

#### 3.4.2. Transcriptomic Analysis of TCA Cycle

In the analysis of gene transcription activity in the TCA cycle (Figure 5), most activities in WG+HLMU medium were generally higher than those in YG+HLMU medium. In particular, the levels of unique genes of *FUM1* and a pair of *PYC1* and *PYC2* in WG+HLMU medium, which could control metabolic reactions, were significantly higher: 5.41 (*p* < 0.05),11.6 (*p* < 0.05), and 736 (tendency existed) times higher than those in YG+HLMU medium. On the other hand, the levels of unique genes of *MDH1* in mitochondrion in peroxisome in WG+HLMU medium were conversely 0.352 times lower than those in YG+HLMU medium.

#### 3.4.3. Transcriptomic Analysis of Pentose Phosphate Pathway and Synthetic Pathway of Amino Acids

According to the analyses of the gene transcription levels in aromatic amino acid synthesis pathway extending from the pentose phosphate pathway (Figure 6a), the levels relating to the metabolism of amino acids at the entrance of the flow of aromatic ring residues such as *ARO3*, *ARO4*, and *ARO1* in WG+HLMU medium were significantly lower (0.0442 (tendency existed), 0.0164 (*p* < 0.01), and 0.0710 (*p* < 0.01) times) than those in YG+HLMU medium; the levels relating to the metabolism of amino acids at the entrance of the flow of histidine synthesis such as *HIS1*, *HIS4*, *HIS6*, and *HIS7* in WG+HLMU medium were significantly lower (0.0372 (tendency existed), 0.0619 (*p* < 0.01), 0.147 (*p* < 0.001), and 0.151 (*p* < 0.01) times) than those in YG+HLMU medium. On the other hand, the transcription levels of *TKL1* and *TKL2* involved in the metabolic flow to glycolysis in WG+HLMU medium were 22.2 and 8.14 times higher than those in YG+HLMU medium, respectively.

The gene transcription levels of the amino acid synthesis pathway extending from pyruvate were analyzed (Figure 6b). When using wheat bran instead of YNB, the transcription levels of unique enzymes *SER1* and *SER2* in WG+HLMU medium were 0.0926 and 0.570 times lower than those in YG+HLMU medium for serine synthesis from 3-phospho-d-glycerate. On the other hand, although the transcription level of *ILV1* in WG+HLMU medium was equivalent to that in YG+HLMU medium, the level of *CHA1* in WG+HLMU medium was significantly 1028 (tendency existed) times higher than that in YG+HLMU medium. For methionine metabolism from serine, the transcription levels of metabolic flow-specific enzymes *SAM2* in WG+HLMU medium were 0.228 times lower than those in YG+HLMU medium. In contrast to these, in the pathway from serine to threonine, the transcription levels of unique genes *GLY1* and *MET2* in WG+HLMU medium were 2.40 and 5.10 times higher than those in YG+HLMU medium. Additionally, in tryptophan synthesis from serine, the unique transcription level of *TRP5* in WG+HLMU medium was 0.145 times lower than that in YG+HLMU medium. Regarding leucine synthesis from 2-oxoisovalerate, the transcription levels of *LEU4* and *LEU9* as unique enzyme genes in WG+HLMU medium were 0.134 and 0.244 -times lower than those in YG+HLMU medium, respectively.

The gene transcription levels in the amino acid synthesis pathway extending from the pyruvate and TCA cycles were analyzed (Figure 6c). Between pyruvate and oxaloacetate, the transcription levels of isoenzymes *PYC1* and *PYC2* in WG+HLMU medium were 11.6 and 736 times higher than those in YG+HLMU medium; between oxaloacetate and aspartate, the levels of isoenzymes *AAT1* and *AAT2* in WG+HLMU medium were also 2.66 and 3.68 times higher than those in YG+HLMU medium. On the other hand, between oxaloacetate and homoserine, the transcription level of the unique enzyme *HOM3* in WG+HLMU medium was 15.0 times higher than that in YG+HLMU medium, whereas that of *HOM2* in WG+HLMU medium was 0.0241 times lower than that in YG+HLMU medium. Between oxaloacetate and 2-oxoglutarate, the related transcription levels in WG+HLMU medium tended to increase towards those in YG+HLMU medium. The metabolite 2-oxoglutarate is an important intermediate contact between the glutamate pathway and lysine pathway. Therefore, the transcription levels relating those pathways were analyzed. (1) Between 2-oxoglutarate and glutamate, the transcription levels of four isozymes were switched, meaning that *AAT1*, *AAT2,* and *ALT2* in WG+HLMU medium were 2.66, 3.68 and 2.82 times higher than those in YG+HLMU medium; *ALT1* was 0.0997 times lower than that in YG+HLMU medium. Additionally, between glutamate and glutamine, the level of *GLT1* in WG+HLMU medium was 9.55 times higher than that in YG+HLMU medium and the level of *GLN1* in WG+HLMU medium was 0.479 times lower than that in YG+HLMU medium. Ornithine is an important intermediate metabolite for synthesizing arginine and proline. Regarding the transcription levels of genes related to the metabolism, the level of *CAR1* in WG+HLMU medium was 0.258 times lower than that in YG+HLMU medium, while the level of *PRO3* was 4.95 times higher than that in YG+HLMU medium. (2) In addition, regarding lysine synthesis, the transcription levels of *LYS2* and *LYS9* in WG+HLMU medium were 4.83 and 7.48 times higher than those in YG+HLMU medium, respectively. On the other hand, the transcription level of *ARO8* was 0.124 times lower than that in YG+HLMU medium.

## 4. Discussion

The assimilation activities of *S. cerevisiae* were evaluated through analysis of its growth in media containing wheat bran as the nutrient component (Figure 1 and Table 2). In order to discuss the values relating to growth, the values in this study should be evaluated relative to the data in previous reports. According to a previous report, the *µ*_max_ value was generally 0.34 ± 0.01 in 250-mL flasks containing 50 mL of YG+HLMU medium at 30 °C [38]. In this study, the *µ*_max_ value in 200 µL of YG+HLMU medium at 23 °C was 0.407 ± 0.035; however, the culturing conditions of volume and temperature were different from the previous report [38]. These conditions should be the same to evaluate the growth property, such that BY4741 was cultivated in 50 mL of YG+HLMU medium at 30 °C, resulting in obtaining the *µ*_max_. The *µ*_max_ value at 30 °C was increased compared to previously reported data [38] and BY4741 in this study showed the same growth property as BY4741 in another laboratory. The specific growth rate of yeast could be normally depressed 0.6~0.7 times with a decrease in the temperature to 23~24 °C from 30 °C [39]; conversely, it could be enhanced by scaling down culturing volumes, with the result that the balanced *µ*_max_ in 200 µL at 23 °C in this study was slightly increased compared to that in 50 mL at 30 °C in this study. To evaluate the use of wheat bran as the nutrient, both the values of *µ*_max_ and OD_600_ of BY4741 in WG+HLMU medium and YG+HLMU medium were evaluated, showing significant differences, and we can therefore reject the null hypothesis. Thus, although BY4741 could use wheat bran instead of YNB as the nutrient for its growth, the strain grew more vigorously in YG+HLMU medium rather than in WG+HLMU medium. Additionally, the fact that the cell density at 24 h in WG+HLMU medium shown by the OD_600_ was 0.83 times lower than that in YG+HLMU medium could reinforce the conclusion that YNB is a better nutrient than the wheat bran for its growth. As per the composition of the wheat bran shown in Table 3, the protein concentration was an adequate supply of nitrogen source for its growth. The ions of Na, Fe, Ca, Cu, and Zn, which are essential metals for growth [32,33,34,35,36], showed as N.D. in the elemental analysis results; however, the growth of BY4741 was not significantly depressed, meaning that these ions were present at concentrations below the detection limit. On the other hand, although the maximum cell density in W+HLMU medium was 0.43 times lower than that in WG+HLMU medium, growth in W+HLMU medium was shown, strongly suggesting that the wheat bran contained a carbohydrate source.

In the case of BY4741 culturing in WG-group media without one of histidine, methionine, or uracil as the essential nutrients, there were no significant differences in cell growth rates (Figure 2a). Compared to WG+HLMU medium, the growth rates in WG-group medium without one of histidine, methionine, or uracil as the essential nutrients were lower than those in WG+HLMU medium; growth was observed in WG-group medium without one of methionine, histidine, or uracil. Therefore, although the essential nutrient forms in the wheat bran were not convenient, the wheat bran contained enough methionine, histidine, and uracil to allow BY4741 to grow. On the other hand, in the case of BY4741 culturing in WG+HMU medium, even on an agarose plate of WG+HMU medium, no cell proliferation was observed. This indicated that the wheat bran did not contain enough leucine to grow. In addition, the result in Figure 2b was that BY4741 and BY4741/pATP425 could be controlled in WG+HMU medium as a selective medium. It was of great significance industrially that the wheat bran, which is significantly cheaper than YNB, could be used as a restricted medium.

The assimilation activity of BY4741 in WG+HLMU medium was evaluated through glucose consumption (Figure 3; Table 4). According to the above analyses, BY4741 cultured in WG+HLMU medium consumed glucose only at a level 60.6% of that in YG+HLMU medium in 24 h, revealing that the efficiency of glucose consumption by BY4741 was decreased in WG+HLMU medium compared to YG+HLMU medium. It is well known that the glucose consumption rate of BY4741 differs depending on the nutritional source [40], and it was shown that wheat bran was no better than the optimized YNB in terms of glucose. However, there was no significant difference in the conversion ratio (theoretical yield) of ethanol from glucose by BY4741 between 63.0 ± 7.2% in WG+HLMU medium and 62.5 ± 8.2% in YG+HLMU medium, and there was no significant difference in ethanol production, even though the efficiency of ethanol production remained the same, meaning that there were no major changes in the metabolic response. There was a change in the efficiency of the cell production from glucose, indicating that the cell response was significant. According to Vieira et al., 47 wt% of the cell weight of the *S. cerevisiae* is carbon [30]. Thus, in this study, in WG+HLMU medium, 47 wt% of 0.600 ± 0.003 g∙L^−1^ of the cell was 0.28 g∙L^−1^ of the carbon as the component of the cells, and 0.705 g∙L^−1^ of glucose was used for cell production, meaning that 7.37 ± 0.04 wt% of glucose was assessed for use in cell production. On the other hand, in YG+HLMU medium, 47 wt% of 0.659 ± 0.021 g∙L^−1^ of the cell was 0.31 g∙L^−1^ of the carbon as the component of the cells, and 0.774 g∙L^−1^ of glucose was used for cell production, meaning that 4.90 ± 0.04 wt% of glucose was assessed for use in cell production. Therefore, the fact that there was a significant difference in cell production strongly suggests that intracellular metabolism, including glucose intaking, could change dramatically. In order to produce substances from the wheat bran resource as a raw material by the use of the intracellular metabolic flow of the BY strain, the evaluation of the intracellular response to the raw material was highly important. Therefore, the attempt was made to analyze and evaluate how BY4741 responded to the use of wheat bran.

The transcriptomic analyses were performed to evaluate the detailed cell responses (Figure 4, Figure 5 and Figure 6). In the case of using wheat bran instead of YNB, the metabolic activities in the upstream glycolysis were possibly enhanced because overall the gene transcription levels were increased (Figure 4a). On the other hand, the transcription levels of *FBA1*/*LOT1*, *TDH1*/*GLD3*, *TDH2*/*GLD2,* and *TDH3*/*GLD1* at the borderline of preparatory and pay-off in glycolysis were depressed in WG+HLMU medium, strongly indicating that BY4741 could activate other metabolic pathways instead of the Embden-Meyerhof glycolysis pathway. Ralser et al. explained that eucaryotic cells could inactive TDH functions as a metabolic switch for rerouting the carbohydrate flux to counteract environmental changes [41], suggesting that *S. cerevisiae* cells tried to shift metabolic flow from glucose to the pentose phosphate pathway via β-d-fructose-6P by replacing YNB with wheat bran. Therefore, a detailed evaluation of the transcription levels in the pentose phosphate pathway was profoundly desired.

When wheat bran was used instead of YNB, the gene transcription levels of *PCK1*/*JPM2*/*PPC1* were enhanced, suggesting that carbon flow could be introduced from the TCA cycle to phosphoenolpyruvate-mediated glycolysis (Figure 4b). Rintze et al. indicated that PCK1/JPM2/PPC1 could perform as a decarboxylase controlling the reactions related to gluconeogenesis, and the transcription of these genes could also be induced by gluconeogenic substrates [42]. The strain might also induce gluconeogenesis when bran was used. Under the medium-condition using wheat bran instead of YNB, *S. cerevisiae* could be about to begin carbon source starvation at the evaluation timing. Thus, the strain can improve growth and production by increasing the concentration of glucose to avoid carbon source starvation. In addition, the increase in the transcription levels of the isoenzymes *ACS1/FUN44* and *ACS2* indicated the influx into the TCA cycle via acetate. This may indicate that the cells were trying to take in the previously produced ethanol as a carbon source. In fact, in 24 h fermentation, the concentration of ethanol in WG+HLMU medium was 69.5 wt% compared to that in YG+HLMU medium. To date, there have been many reports that BY4741 utilizes the ethanol produced by itself as a carbon source [43], and this acetic acid-mediated metabolic flow from ethanol could be likewise enhanced in the case of using wheat bran. In addition, since BY4741 is also able to regenerate NADH from NAD^+^ in the metabolic pathway from acetaldehyde to acetic acid [44], the strain could use this alternative pathway instead of the pathway regulated by the repressed gene transcriptions of *TDH1*/*GLD3*, *TDH2*/*GLD2*, and *TDH3*/*GLD1*, which similarly control NADH production. Regarding ethanol production, the *ADH1* transcription level decreased significantly. Although no significant difference in ethanol production efficiency was confirmed in this study, the significant difference between the *ADH* transcription levels in WG+HLMU medium and YG+HLMU medium could affect metabolism because of ADH1′s activity of regenerating NADH from NAD^+^ [45,46].

In the case of using wheat bran instead of YNB as the medium component, the gene transcription levels of *PYC1* and *PYC2* were high, with significant differences (Figure 5). Similarly, the transcription levels of *CIT1* and *CIT3* remained high, suggesting that carbon flow via pyruvate into the TCA cycle was induced. In particular, significantly high transcription levels of *PYC1* and *PYC2* possibly indicate that the cells wanted to introduce a carbon source not only into the TCA cycle but also into the amino acid synthesis pathway via aspartate. In terms of the amino acid synthesis pathway, by using wheat bran instead of YNB, the transcription levels around 2-oxoglutarate tended to be activated as a whole, indicating the possibility that amino acid synthesis such as glutamic acid was activated. To clarify the above, evaluation of the transcription levels of these pathways would be highly desirable.

In the case of using wheat bran as an alternative nutrient to YNB, the gene transcription levels of isozyme genes *TKL* were increased (Figure 6a). The result showed the possibility that the metabolic activity from fructose-6 phosphate to glycelaldehyde-3 phosphate was enhanced. The enhancement of the pentose phosphate pathway via ribose 5-phosphate was possibly reinforced to produce NADPH, which was consumed in the biosynthetic pathways of branched-chain amino acids such as glutamate and proline. So far, the cells are known to enhance the pentose phosphate pathway to produce NADPH [47], possibly indicating that culturing in wheat bran media promoted the synthesis of these amino acids using the pentose phosphate pathway. In particular, as shown in Figure 4a, in WG+HLMU medium towards in YG+HLMU medium, the transcription levels of *FBA1*/*LOT1*, *TDH1*/*GLD3*, *TDH2*/*GLD2,* and *TDH3*/*GLD1* corresponding to the enzymes around glyceraldehyde in glycolysis were depressed; the transcription levels between glyceraldehyde-3 phosphate and glycerol were increased/maintained; the levels of all genes between glycerol and 3-phospho-d-glycerate were, conversely, enhanced. Those results indicated the possibility that glucose could flow to 3-phospho-d-glycerate via the pentose phosphate pathway and glycerol producing pathway rather than via fructose-6 phosphate. Regarding the production of tyrosine and phenylalanine, the transcription levels of *ARO3*, *ARO4,* and *ARO1* should be considered to be controlling factors on the metabolic pathway. ARO3 and ARO4 are generally known to be feedback-inhibited by tyrosine and phenylalanine. Therefore, as Braus suggested that the transcription levels of these genes were regulated by the presence of substrates [17,48], the transcription levels of *ARO3* and *ARO4* could also be sensitively affected by the presence of tyrosine and phenylalanine in the medium. Additionally, the depressed transcription level of ARO1, an enzyme which controls the unique pathway between d-erythrose 4-phosphate and 5-O-3-phosphoshikimate via shikimate, could also be related to the decrease in activity for the production of tyrosine and phenylalanine.

In the case of the use of wheat bran as an alternative to YNB, regarding the synthesis from 3-phospho-d-glycerate to serine, the depressed transcription levels of *SER1* and *SER2* and the enhanced levels of *ILV1* and *CHA1* could indicate the reinforcement of the synthesis via pyruvate (Figure 6b). Pyruvate serves as a starting point for synthesizing multiple amino acids, so that the cells might have prioritized the metabolic flow to pyruvate. Serine biosynthesized from pyruvate is an important intermediate metabolite. In WG+HLMU medium, with the regard to the synthesis from serine to threonine, the synthesis was mainly performed in the pathway via cystathionine and homoserine with the repression of the transcription levels of *TRP5* and *SAM2* in tryptophane synthesis. The fact that the transcription level of *TRP5*, which is a gene of an enzyme controlling the pathway from serine to tryptophane, was depressed could have the effect of decreasing runoff of intermediate metabolites on the pathway to cystathionine and homoserine. Similarly, SAM2, which does not receive negative feedback control depended on the methionine quantity unlike SAM1, controls methionine metabolism [49] so that the transcription level of this could also have the effect of decreasing runoff on the pathway. Homoserine is an important intermediate metabolite for producing threonine and methionine in the cell [50], meaning that BY4741 might be satisfied by taking the roundabout route. In addition, with regard to leucine biosynthesis from 2-oxoisovalerate, in WG+HLMU medium, the cells decreased the transcription activities of *LEU4* and *LEU9* as the unique genes in the pathway. As with the biosynthesis of methionine and tryptophan, the cells might control leucine synthesis.

In the case of the use of wheat bran instead of YNB, the enhanced gene transcription level of *PYC* possibly indicated a reinforcement of the metabolic flow to oxalacetate as described with respect to Figure 5. Oxaloacetate is not only an intermediate metabolite in the TCA cycle, but also deeply related to the synthesis of the various amino acids via 2-oxoglutarate [51]. From the viewpoint of oxaloacetate as the starting point, the transcription levels of *AAT1* and *AAT2* were greatly enhanced, while that of *HOM2* was significantly decreased, indicating the promotion to synthesize asparagine and aspartic acid; the transcription levels in the metabolic pathway toward 2-oxoglutarate showed a tendency to be improved, suggesting the possibility that amino acid synthesis originating from 2-oxoglutarate was also promoted (Figure 6c). Between 2-oxoglutarate and glutamate, the cells tended to maintain metabolic flow even though the transcription levels of the isoenzyme gene were switched. In addition, glutamate is an important intermediate metabolite leading to arginine and proline [51]; the level of *GLN1* related to the flow from glutamate to glutamine decreased, and the level of *GLT1* related to the flow from glutamine to glutamate increased. Thus, BY4741 tended to maintain glutamate as the intermediate metabolite in the use of wheat bran. Furthermore, due to the decrease in the levels of *ARG1* and *CAR1* and the increase in that of *PRO3*, ornithine possibly flowed not to arginine but to proline. In addition, lysine synthesis from 2-oxoglutarate might also be regulated, since the transcription level of *ARO8* was decreased although those of *LYS2* and *LYS9* were increased.

The evaluation of the gene transcription levels could not directly indicate the effects on the metabolic flows since the gene translation activity and enzyme activity after the gene transcription were influenced by circumstance situation; however, the activity evaluation allowed us to evaluate how cells wanted to respond. Then, the cell responses in the medium containing wheat bran instead of YNB were shown by evaluating the changes of the gene transcription levels. The aim of this study was the evaluation of the cell responses when wheat bran was used in place of YNB; our study reached the goal of revealing how cells attempt to respond from the viewpoint of transcriptome analysis. The results reported in this study are beneficial in the academic aspect because they could be useful to detect which points should be overexpressed by genetic engineering when the mutants are to be prepared to produce value-added substances in wheat bran medium.

## 5. Conclusions

In this study, the cell responses of *S. cerevisiae* BY4741 strain in the medium containing wheat bran were evaluated by analyzing cell growth and the production of cells and ethanol, and the gene transcription levels. Herein, wheat bran could be used as an option as the nutrient resource for *S. cerevisiae* cultivation not only in the laboratory but also in industry. The possibility of using wheat bran as the nutrient resource restricted on leucine was demonstrated by showing experimental data on cultivation, suggesting the availability of other types of biomass nutrients such as sugarcane juice and molasses. These results revealed that BY4741 grew in the medium containing the wheat bran as a nutrient source instead of YNB; the strain possibly switched the carbon metabolic flows due to replacing YNB with wheat bran according to the difference of the conversion ratio of cells; in fact, the strain showed different transcription levels in glycolysis, the pentose phosphate pathway, the TCA cycle and the amino acid synthesis pathway according to the transcriptomic analyses. Wheat bran is a useful medium component for BY4741; however, when a BY4741 strain whose intracellular metabolism has been modified by genetic engineering is used, deep consideration should be given to whether the responses of the transcription levels in the mutants will be suitable under conditions using wheat. Therefore, our research could give useful information for using the metabolic pathway of BY strain from the viewpoint of genetic engineering, meaning that the knowledge in this study will be able to be used in the fields of the food production and food recycling.

## Figures and Tables

**Table 2:** Growth properties of BY4741 in each medium.

Strain	Medium Name	Time Range (h)			Maximum Specific Growth Rate (µ_max_, h^−1^)			Doubling Time (h)			Cite
BY4741	WG+HLMU medium	6.0	~	9.0	0.277	±	0.002	2.50	±	0.01	Figure 1a
	YG+HLMU medium	10.0	~	11.5	0.407	±	0.035	1.71	±	0.15	Figure 1a
	G+HLMU medium		−		N.D.			N.D.			
	W+HLMU medium	3.0	~	9.0	0.131	±	0.004	5.29	±	0.15	Figure 1b
	Y+HLMU medium		−		N.D.			N.D.			
	HLMU medium		−		N.D.			N.D.			
BY4741	WG+HMU medium		−		N.D.			N.D.			Figure 2a
	WG+LMU medium	14.0	~	22.0	0.0536	±	0.0019	12.9	±	0.5	
	WG+HLU medium	4.0	~	22.0	0.0521	±	0.0007	13.3	±	0.2	
	WG+HLM medium	10.0	~	22.0	0.0484	±	0.0022	14.6	±	0.7	
BY4741/pATP425	WG+HMU medium	6.0	~	16.0	0.0901	±	0.0048	7.71	±	0.39	Figure 2b
BY4741	WG+HMU medium		−		N.D.			N.D.			

**Table 3:** Composition of wheat bran and YNB.

	Main Components (g·L^−1^)				Minerals (mg·L^−1^)								
	Carbohydrate		Protein	Lipid	Na	P	Fe	Ca	K	Mg	Cu	Zn	Mn
	Total	Glucose											
Wheat bran	15.1	1.7	6.1	0.0	˂10	69	˂1	˂10	98	26	˂0.1	˂0.5	0.7
YNB	0.0	0.0	0.0	0.0	40	227	0.07	36	287	101	0.02	0.2	0.1

**Table 4:** Activity of glucose use in WG+HLMU and YG+HLMU at 24 h.

	Glucose Consumption			Cell Production							Ethanol Production						
	Actual Value (g∙L^−1^)			Actual Value (g∙L^−1^)				Conversion Ratio (%)			Actual Value (g∙L^−1^)				Conversion Ratio (%)		
WG+HLMU medium	9.57	±	0.69	0.600	±	0.003		7.31	±	0.04	3.08	±	0.35		63.0	±	7.2
YG+HLMU medium	15.8	±	0.16	0.659	±	0.021		4.90	±	0.16	5.05	±	0.66		62.5	±	8.2

## Data Availability

Not applicable.

## Supplementary Materials

The following supporting information can be downloaded at https://www.mdpi.com/article/10.3390/microorganisms11112674/s1, Table S1: quantitative PCR primers.
