# Supplementary material for: Evaluation of Cell Responses of Saccharomyces cerevisiae under Cultivation Using Wheat Bran as a Nutrient Resource by Analyses of Growth Activities and Comprehensive Gene Transcription Levels
Source: Microorganisms. 2023 Oct 31;11(11):2674. doi: 10.3390/microorganisms11112674 (PMC10673363; doi:10.3390/microorganisms11112674)
Supplement: Supplementary file 1 [file microorganisms-11-02674-s001.zip › microorganisms-2683954-supplementary.pdf]

Supplemental Table S1. quantitative PCR primers

Gene		Primer sequences (5'→3')
TDH2	F	cggtagatacgctggtgaagtttc
	R	tggaagatggagcagtgataacaac
TDH3	F	tttcccacgatgacaagcac
	R	gaagcccatggcaagttagc
RDN18	F	aactcaccaggtccagacacaataagg
	R	aaggtctcgttcgttatcgcaattaagc
KRE11	F	aactggttctgttacccaaatcaactcaac
	R	aacgcttcaatgtgacttctgtttccc
SGA1	F	tccaaacggatatttcctgggtggtactgag
	R	gcatgatctattgtgtttacattagcgggtag
PGM1	F	tgatcctgccaaagcatacg
	R	ttgcgctgtttcgctaagaa
PGM2	F	gcctggtacctctggtttgc
	R	ccatcaccaccgacaacaag
PRM15	F	aagaggtcaccgctctctgc
	R	accagcagtgccaaactgaa
PGL1	F	acttcacccaaaccccattg
	R	tcgaatggagcaaccaaatg
HXK	F	aggggaggtagttgcaatgg
	R	tccggtgaaaggaagattgg
HXK1	F	attccaatgattcccggttg
	R	ccgctcaacttgaccaacac
HXK2	F	aaattttgcgtttggccttg
	R	tgtccatgacgaaaggcttg
GLK1	F	gccggtcatcggatgtattt
	R	gacccccattcgacattgat
GAL10	F	tgaaatggcagaccgagttg
	R	gcctcgacaccccttaactg
PFK1	F	actcccgtgcctttgttgtt
	R	atcggcaccggtagcaatac
FBP1	F	cgcctcaaaaggccatctac
	R	tgtctgcttggggttgtttg
FBA1	F	ggctttgcacccaatctctc
	R	aaagcgatgtcaccagcgta
TPI1	F	ggtaccggtttggctgctac
	R	gctaccgttagcggaaccac
TDH1	F	atggtccatcccacaaggac
	R	ggcaagaccttaccgacagc
PGK1	F	attggtggtggtgacactgc
	R	agcaacacctggcaattcct
TDA10	F	gcgcaacccagaaatcaaat
	R	gcacttgttcgtcggtcatc
ALD2	F	tcattcccccaaccatcttc
	R	gcgagcccgtagcaagtatc
ALD3	F	tcattcccccaaccatcttc
	R	gcgagcccgtagcaagtatc
ALD4	F	aaccaacggggttgttcatc
	R	gcctcttccacatcgtcctc
ALD5	F	aaggtcgctgccgatactgt
	R	cgcagcaaacttcaccagag
ALD6	F	tgaatgggctacccaagacc
	R	gtaacatccccacgggctaa
HFD1	F	gcgcacaaaaggaaaagctc
	R	ggcgtaacccccacttgtta
GCY1	F	tggcagtcgaaagagaacga
	R	ttgatggcttgaccgacttg
DAK1	F	aggcggtaaaagctgcagag
	R	tgaatcgccgacatacgaag
DAK2	F	ctgatcgatgctctgcaacc
	R	ttcggcaccatcataagcag
GUT1	F	atggcgcactgacgactcta
	R	tagccattggaccacagcac
GPP1	F	catcgaagttccaggtgctg
	R	tgtcacgggtaccagaggtg
GPP2	F	cattgaagtcccaggtgcag
	R	atcacgggtaccggaagttg
GPT2	F	accacccggaaatcatcaag
	R	tccgggatttccttgatttg
SCT1	F	tatggctcttggttgcatgg
	R	atggggtcaccgaattcaac

Gene		Primer sequences (5'→3')
PHM8	F	ttgcaaagtgggttgtcgag
	R	atggataacatggcccatcc
GPM2	F	gcaagcacaccatccaatgt
	R	gcgtcaatccaaccacagaa
ENO1	F	tgacgaaggtggtgttgctc
	R	cttaccgtcgtgaccagcag
CDC19	F	cgatttgccagctttgtctg
	R	aaaacatcgttggcggttct
PCK1	F	cagaatcaaagtccgcgttg
	R	ctgaccagcgttccagacag
PDA1	F	cttcattcaaacgccaacca
	R	tgtcctcaggggccttctta
PDB1	F	ccgctttgaagggtttgaag
	R	tttgcagcggaattgacaac
THI3	F	gcaagctctttggggatctg
	R	ttgcggggaatttatccttg
PDC1	F	catgatcagatggggcttga
	R	gagcctttggaccgtgaatc
PDC5	F	gatcagaaacgccaccttcc
	R	ctgggacagcaacaggtttg
PDC6	F	gcctcacgcagagtacaacg
	R	tggcgatcttgtgattttcg
LPD1	F	gccacgggctctgaagttac
	R	aacccatttccaatccgatg
LAT1	F	ggctaagagggtgccagatg
	R	gtggcaacagcgactgagac
ACS1	F	ttttatgttgcgccaactgc
	R	acccaagcaacgcaaagatt
ACS2	F	ccttgggtaccgcctcaata
	R	tgggtagccttgtgacgttg
ADH1	F	cgttaagggctggaagatcg
	R	ccagacaagtcagcgtgagg
ADH2	F	cgttaagggctggaagatcg
	R	ccagacaagtcagcgtgagg
ADH3	F	aagccgccaaaattcaacag
	R	accagagatggcaacccagt
ADH4	F	gctgttgctgtcaacgatcc
	R	gaggcggtggaaacataagc
ADH5	F	tcgatggtggtaatgccaag
	R	actccatgagaaccgccatt
ADH6	F	tccatcacatttggctgctc
	R	tttttacctggaccgcaacc
ADH7	F	ggcgatcatgacgttgatgt
	R	tgggactggaccccaattac
SFA1	F	aatgactgatgggggtctgg
	R	ggcagccacaccaatgataa
CIT1	F	acccatggccaaatgttgat
	R	ggagcaccaacagccctatc
CIT2	F	attcggatcacgaaggtggt
	R	caaacctgatgcaagggaca
CIT3	F	tgcgttgagtgacccgtatc
	R	cttgtgctgctaacccatgc
ACO1	F	tgccatcaagagacccattg
	R	tccagcgtttccacattctg
IDP1	F	atgtgccaccatcactcctg
	R	cgccgagaatgtttctgatg
IDP2	F	tcggccaaatattggaaagc
	R	tagattccaccgcgtcaatg
IDP3	F	ataggccgtcatgcttttgg
	R	gcgatcccaccacttttagg
IDH1	F	atccgtccctggtgtagtgg
	R	ggcgaagtcaaaggcaaatc
IDH2	F	aaggtggtcaccaacccatc
	R	taaagaaccggcgctcaaac
KGD1	F	acccattcccatttgctcag
	R	tctgtgtatgcccacgaacc
KGD2	F	tattccagccgtcaatggtg
	R	ttacgaacgacgggggtaac
LSC1	F	atgggtggtgatgcttttcc
	R	ttcgatttcggccttaccac

Gene		Primer sequences (5'→3')
LSC2	F	ggatttccagcgttcactcc
	R	cctctacctccggtcaatgc
SDH1	F	attcccacgaagtggaatgg
	R	ttggcaccatggacagaaac
SDH2	F	ggaaggccttttgtttggtg
	R	tagcacttggctcgtctgga
SDH3	F	ctccgccttttcaagagcag
	R	ccgctttggtgttcatttca
SDH4	F	caggtggtgttaggggcact
	R	acgacggacaaggcaaagat
SDH9	F	gggtctggctgcctaatcac
	R	ttccatggtgattgctctgg
SHH3	F	ccccgagcatctgtattcgt
	R	cggccattgtggaattcttt
SHH4	F	aaagcagagctcagggttcg
	R	accaagggaactgcgatcag
FUM1	F	aagaatgccattgcctttgg
	R	ggcctgttgaatagccttgg
MDH1	F	tccgtggtcaaggggtttac
	R	atggcgaacaagtcatcacg
MDH2	F	acatggaacgcaaccaaatg
	R	tcgatgcaacgaattccaag
MDH3	F	tggaaagttcgcaccaaatg
	R	ctgcacgtaccaggtcaagg
HIS1	F	agtggttccgtggaggcatc
	R	gtggcttaggacggtggctc
HIS2	F	cgggcaactgcaacgaagaa
	R	gcgccgccataggagtctat
HIS3	F	caccactgaagactgcggga
	R	gcgtacggcctgttcgaaag
HIS4	F	ggtgccaacactgcaacctt
	R	cctccttcttggcaacgcac
HIS5	F	tggtggctctacagctccac
	R	ctggccaactctgctgttgc
HIS6	F	gacggtcgttggattgtggc
	R	ccttcaacgtctgcagcgtg
HIS7	F	agctctaggtgctggggaga
	R	gccactggatgcaatgacgg
PRS1	F	ggtgacgatgaggcagacga
	R	tcgtcttcggagtcgacagc
PRS2	F	ggtgcgaaaagggtggcttc
	R	cgacaagcagcattcgcgaa
PRS3	F	gcttcgaagactgcgtctgc
	R	tgatcacacccggctgttgt
PRS4	F	tggatctccatgcctcccaa
	R	cctgcatcaggcgacaccaa
PRS5	F	tccagagtggttgtggccag
	R	taaccctcgaggcagaagcg
RKI1	F	tcgatgcggatttcggtgaa
	R	tgtttccaccacgcccacta
RPE1	F	agggcatcaaagctgcatgc
	R	ttggcctccaaacccaggtt
TKL1	F	aactcaggtcacccaggtgc
	R	ccagtctgggttggttgggt
TKL2	F	atagcgcaggccaactttgc
	R	cccaattgcagatgtcccgc
NQM1	F	ttggtggaattcggcaccca
	R	agagccttcttcaccgtcgc
TAL1	F	tgccgaggcccaagttactt
	R	aacacctgggtcggcttcac
ARO1	F	ggtgcccatgctgcttttgt
	R	tccagggacaacgtgccatt
ARO2	F	gcggacttcacgtactcgga
	R	ctgaagcgacacggccaatc
ARO3	F	ccatcggcgccagaactact
	R	gttcatgtgcagcggctctc
ARO4	F	ctcaattgcctgccggttcc
	R	tggcgttttcaccgttagcg
ARO7	F	tcaccaattctgccgttgaaga
	R	cgttggtagggtccacacca

Gene		Primer sequences (5'→3')
ARO8	F	accttccccgtgccaattga
	R	ggagcaccaggtgtccagtt
TRP1	F	gcaggtgggacaggtgaact
	R	gcaggtgggacaggtgaact
TRP2	F	gaaggagaaaggcgtggggt
	R	caataccaccgccagcttgc
TRP3	F	gctgaagtcaagcgtgcctc
	R	atgcaccagcctctgcgtat
TRP4	F	atgtccgaggcgactttgct
	R	ccagcaacgcatcgtgtagg
TRP5	F	tacccaggtgtcggtccaga
	R	agcctgagcgtcagtagctg
PHA2	F	cggccgattgcgacaacatt
	R	gcctgcgttctcccttctct
TYR1	F	tggtatccaggcaaagcgact
	R	tccggcagcttacaactcgt
SER1	F	caacatttcggagcagggcc
	R	tggcatccttcgaacggtga
SER2	F	tggtgggtgacggtggtaac
	R	ttctgcacctttggcttggc
SER3	F	gccttgggtactgccagaca
	R	gcgccatccttcatagcagc
SER33	F	tcgctgctatgaaggacggg
	R	gcacctgcaattttgttggcc
CHA1	F	tgattgaaccggcatgtggc
	R	aggagccgccacaagcaata
ILV1	F	tgaagagcgtggcttgacga
	R	cgggaacaaagacagcaccg
MYC1	F	cattgctggctgcggtactg
	R	tccgctagaacgacatggca
MET2	F	agggaggagacacgcaaacc
	R	tggacaccacgctttgacct
MET6	F	acgttgctgctgccctagaa
	R	ccttaacggcagcatcgtgg
MET17	F	aaggtggtgctgctgctttg
	R	caccagtgtgtgccaaacct
CYS3	F	aatcgcttcctcagggctcc
	R	gcgttggcgactttggtgaa
CYS4	F	ttgctggtgctggtactggt
	R	cgaatgggtcagcaccaacg
IRC7	F	ggcaatgttaccaggtgccg
	R	gatttccgagccaagcagcc
STR2	F	atcttcagcggcgactcgaa
	R	cgcatcttcgcaccataggc
STR3	F	ggattacgctccaacccgga
	R	tcgaaggaccccgtttcgaa
SAM1	F	tgtgaaaccgcggcaaagac
	R	agtcgaaacccttggcggaa
SAM2	F	tggtcctcaaggtgacgctg
	R	ttaccggagaaggcaccacc
SHM1	F	tgatcgtggccggtacttcc
	R	ggacaacattggctgccacc
SHM2	F	atcgccgacaaatgtggtgc
	R	agatgggatgacacctgcgg
GLY1	F	acgacttgcggtcagacaca
	R	acagcgtcaccgatagaggc
THR1	F	gtcaggtgcaggcccaacta
	R	tagcaccatcgtaggcaggc
THR4	F	aacacaacgtcggtgctgtt
	R	accgttggtggcttggaaga
ILV2	F	ttacaacgggtgtggggcaa
	R	caacttgagcaccgatggcg
ILV3	F	accggtgacactttggcaga
	R	tttgcaagtgaccgttggcc
ILV5	F	gctgccatcgaagacggttg
	R	gagcggcatcggacaacaag

Gene		Primer sequences (5'→3')
ILV6	F	caagacctcccttgcccaca
	R	acaccgggttcgttttgcac
ALT1	F	ggcagttcaagaagctcgcc
	R	accgggaacagtgcaaatgc
ALT2	F	aaagcgaacgccttggcatc
	R	aagtcccgggtctttgtccg
BAT1	F	ctcgctactggtgccccatt
	R	tcggtgaatgtctggccgaa
BAT2	F	gctgccctgtgggtccttat
	R	ttgtcaccacagcctcctgg
LEU1	F	atcgtccacaccatcggtcc
	R	taccaaaggccagcgaacca
LEU2	F	tgggtttgttgccatctgcg
	R	accgtggcatggttcgtaca
LEU4	F	tggggtgtaggtgtctccga
	R	ccttcgacctcggccaaaga
LEU9	F	gcaacccaagcggcatcttt
	R	ggagacaccaacaccccaca
PYC1	F	agaatgttggggtggtgcca
	R	caccattggcaccacgcaat
PYC2	F	ttcccggtactccaggacct
	R	gccacgtcgtcaccttctct
AAT1	F	gtccgcagtttgggccattt
	R	tacagctagggcgcctgtac
AAT2	F	gcactcttgcgcccataacc
	R	tccagtggcaaacccttggt
HOM2	F	actgcaggtttggttgcacc
	R	tggggagaaaccagcacctg
HOM3	F	gggtcgtgcaaaagttcggt
	R	aagaacgggcggaacaaacg
HOM6	F	cgttatcggtgccggtgttg
	R	agaacgctcagcttcagcca
ASN1	F	acgcattccttccaccccaa
	R	ccgtatgggacttcggccat
ASN2	F	gggccatttctgacgccatg
	R	acagtgtctgccgcagtctt
LYS1	F	cggtgccatcgatctgttgc
	R	aaagggaccaccacgggaag
LYS2	F	gcgggctcactggtggatat
	R	aaccattggcagaggcacct
LYS4	F	acggccctgtgatcttggag
	R	acccaaccttccaagcctcc
LYS9	F	ccaattgcctggaacgaagca
	R	gcctaaccaagcaaacccgg
LYS12	F	ccatgccatggttctgcacc
	R	cagcttcgttgtggcccaag
LYS20	F	gctttggaaggtggtgccag
	R	ttcttgccatgagcccacct
LYS21	F	gctttggaaggtggtgccag
	R	ttcttgccatgagcccacct
ACO2	F	aaagccggcttgaaaccacg
	R	gggccgcatgcatttgctaa
GLT1	F	atgtccagcaccctgtgagg
	R	agccagtgcgtgtacttggt
GLN1	F	accgcttccatgactgcctt
	R	ccttccttggcgacggatct
PRO1	F	caaacccagatgccatgccg
	R	cggtcccaacgtcagaacct
PRO2	F	agggtgtcgactcctctggt
	R	actcaccagaccgtccaagc
PRO3	F	gtgcggtggtgtcctactca
	R	agccgtagcagcatccatgt
ARG1	F	acgctctgaagccagacgtt
	R	tggtttgggcgacgggaata
ARG2	F	gcaccagcgattttgtccca
	R	gttggcgacggcggaattac
ARG3	F	tgctgagagaaacggtgcga
	R	cagcttggcctgtttcgcaa
ARG4	F	ggggtgggagattcactggt
	R	tctgcaagcccgctgtgtat
ARG7	F	tttgcatgttggccaacggt
	R	tcgaaccttcaccatcgcga
ARG8	F	accaagtgaggctcatccgg
	R	cattgacgatggtggcagcg
ARG56	F	tcaaggtgggtggtgccatt
	R	ggctcaattccctgcgcttc
CAR1	F	cgccgccttttccatgtacc
	R	tttgtttctgggtgcacggc
